# Exogenous Stilbenes Improved Tolerance of *Arabidopsis thaliana* to a Shock of Ultraviolet B Radiation

**DOI:** 10.3390/plants10071282

**Published:** 2021-06-24

**Authors:** Zlata V. Ogneva, Vlada V. Volkonskaia, Alexandra S. Dubrovina, Andrey R. Suprun, Olga A. Aleynova, Konstantin V. Kiselev

**Affiliations:** 1 Laboratory of Biotechnology, Federal Scientific Center of the East Asia Terrestrial Biodiversity, Far Eastern Branch of the Russian Academy of Sciences, 690022 Vladivostok, Russia; zlata.v.ogneva@gmail.com (Z.V.O.); volkonskaia.vv@students.dvfu.ru (V.V.V.); dubrovina@biosoil.ru (A.S.D.); suprun.hi@gmail.com (A.R.S.); aleynova@biosoil.ru (O.A.A.); 2 The School of Natural Sciences, Far Eastern Federal University, 690090 Vladivostok, Russia

**Keywords:** plant UV tolerance, piceid, resveratrol, stilbenes, UV-B irradiation

## Abstract

Excessive ultraviolet B (UV-B) irradiation is one of the most serious threats leading to severe crop production losses. It is known that secondary metabolite biosynthesis plays an important role in plant defense and forms a protective shield against excessive UV-B irradiation. The contents of stilbenes and other plant phenolics are known to sharply increase after UV-B irradiation, but there is little direct evidence for the involvement of stilbenes and other plant phenolics in plant UV-B protection. This study showed that foliar application of *trans*-resveratrol (1 and 5 mM) and *trans*-piceid (5 mM) considerably increased tolerance to a shock of UV-B (10 min at 1800 µW cm^−2^ of irradiation intensity) of four-week-old *Arabidopsis thaliana* plants that are naturally incapable of stilbene production. Application of *trans*-resveratrol and *trans*-piceid increased the leaf survival rates by 1–2%. This stilbene-induced improvement in UV-B tolerance was higher than after foliar application of the stilbene precursors, *p-*coumaric and *trans*-cinnamic acids (only 1–3%), but less than that after treatment with octocrylene (19–24%), a widely used UV-B absorber. Plant treatment with *trans*-resveratrol increased expression of antioxidant and stress-inducible genes in *A.* *thaliana* plants and decreased expression of DNA repair genes. This study directly demonstrates an important positive role of stilbenes in plant tolerance to excessive UV-B irradiation, and offers a new approach for plant UV-B protection.

## 1. Introduction

Due to their sessile nature, plants are exposed to a great variety of stressful conditions such as high soil salinity, heat, cold, drought, and ultraviolet (UV) radiation. The solar UV component of solar light is composed of three parts: UV-A (400 nm–320 nm), UV-B (320 nm–290 nm), and UV-C (290 nm–200 nm). UV-C is the most harmful to plants and is entirely absorbed by stratospheric ozone, oxygen, and other atmospheric gases [1]. Thus, plants are normally exposed to only UV-A and UV-B radiation. UV-A radiation is the least energetic and damaging, but excessive UV-B irradiation is a serious threat leading to severe crop production losses [2]. UV-B radiation is known for its negative effects on plant growth and development, photosynthetic apparatus, as well as DNA and chloroplast damage [3,4,5]. Depletion of the ozone layer leads to an increase in the UV-B radiation reaching the plant surface. This raises the interest in studying the mechanisms and methods of protecting plants from excessive UV-B radiation.

Plants developed a number of protective strategies to reduce the negative effects of UV-B, including morphological and physiological processes. For example, changes in the leaf anatomy (especially leaf thickness, size, and pigmentation) serve to protect plant photosynthetic apparatus from excessive UV-B [2,6]. However, such mechanisms usually reduce the amount of photosynthetically active radiation that is absorbed by the leaves and, therefore, reduce photosynthesis. In addition, UV-B increases the density of leaf trichomes, increases axillary branching, and decreases stem elongation [7]. Another approach is the synthesis of polyamines, proteins with antioxidant function, DNA repair proteins, ascorbate, glutathione, and a wide range of plant-specific secondary metabolites [8,9].

The contents of UV-absorbing phenolic secondary metabolites such as various flavonoids (e.g., flavones, isoflavonoids, phenolic acids, or anthocyanins), stilbenes, or carotenoids are highly increased after treatment with ultraviolet light [10,11,12,13,14]. Plant phenolics are known to act as UV-B absorbers preventing UV-B from entering the leaf mesophyll cells, and to detoxify reactive oxygen species (ROS) or reduce their formation [15,16]. There is also direct evidence for the strong role of plant phenolics in UV-B protection. For example, mutants of Arabidopsis defective in the ability of flavonoid biosynthesis have enhanced UV-B injury and oxidative damage [17].

Plant stilbenes have been the subject of intensive research due to their neuroprotective, cardioprotective, anticancer properties [18,19]. They have important roles in plant disease resistance including anti-fungal and anti-bacterial activities [20]. *Trans*-resveratrol (or *t*-resveratrol) is the most well-known stilbene because it is most commonly found in nature and has highly valuable health-promoting properties and biological activities [18,21].

Regarding stilbenes and ultraviolet radiation, the findings available in the literature indicate that stilbenes, as well as other plant phenolics, are implicated in plant protection against the ultraviolet damaging effects [22,23]. Multiple studies demonstrated that UV-C irradiation dramatically increased stilbene production [22,23]. A limited number of reports showed UV-B stimulating effects on stilbene accumulation, although it was less extensive than after UV-C treatments [24,25,26,27]. The stilbene biosynthesis activation was accompanied by enhanced expression of stilbene biosynthesis-related genes and their protein levels [26,28]. The molecular mechanisms of stilbene biosynthesis activation in response to UV radiation and their specific contribution to plant photoprotection remain poorly understood [22,23]. According to our recent data, transgenic plants of *A. thaliana* overexpressing a stilbene synthase (*STS*) gene and accumulating increased stilbene amounts exhibited improved resistance to UV-B and UV-C radiation in comparison with plants transformed with a control vector [29]. Transgenic grapevines overexpressing the *VpSTS29* gene showed a decreased H_2_O_2_ content and an altered expression of genes related to redox processes, stilbene biosynthesis, and light stimulus [30]. To the best of our knowledge, there are no other studies providing direct evidence for the UV-B protective effect of stilbenes. Overall, there is relatively little evidence for the involvement of stilbenes in plant UV-B protection and for the mechanisms responsible for this effect.

Plant stilbenes and other plant phenolic secondary metabolites have gained considerable attention and experimental evidence for their animal and human skin photoprotective effects against ultraviolet radiation [31,32]. It is reasonable to propose that plant stilbenes as well other plant-derived phenolics can offer plants photoprotection against excessive ultraviolet radiation. In this study, we investigated whether foliar stilbene application can be used for increased plant survival under UV-B irradiation. This study is the first to evaluate the plant UV-B protective effect of exogenous stilbenes (*t*-resveratrol, *t*-piceid) and stilbene precursors (*p*-coumaric acid, *t*-cinnamic acid) applied to the foliar surface of *Arabidopsis thaliana* L. after UV-B irradiation.

## 2. Results

### 2.1. The Effect of Exogenous Stilbenes, Stilbene Precursors and Octocrylene on A. thaliana Growth

Aqueous solutions of stilbenes (*t*-resveratrol, *t*-piceid), stilbene precursors (*p*-coumaric acid, *t*-cinnamic acid), and octocrylene at the concentrations of 1 and 5 mM were sprayed with a 2 mL atomizer polypropylene vials onto the adaxial and abaxial leaf surface of the four-week-old rosettes of *A. thaliana*. We then investigated the effects of the stilbenes, stilbene precursors, and octocrylene on the leaf number and fresh biomass accumulation of *A. thaliana* (Table 1). Octocrylene neutralizes ultraviolet radiation in the most damaging range (approximately 280 nm–300 nm), showing peaks of the most active adsorption (280 nm and 295 nm). We used octocrylene as a control compound with recognized positive UV-protective properties. None of the treatments considerably reduced the number of leaves and fresh weight of the *A. thaliana* rosettes (Table 1). On the contrary, treatment with *p-*coumaric acid increased the *A. thaliana* rosette fresh weight by 1.5-fold (Table 1).

### 2.2. The Effect of Exogenous Stilbenes, Stilbene Precursors and Octocrylene on A. thaliana Leaf Survival after the UV-B Treatment

**Figure 1:**
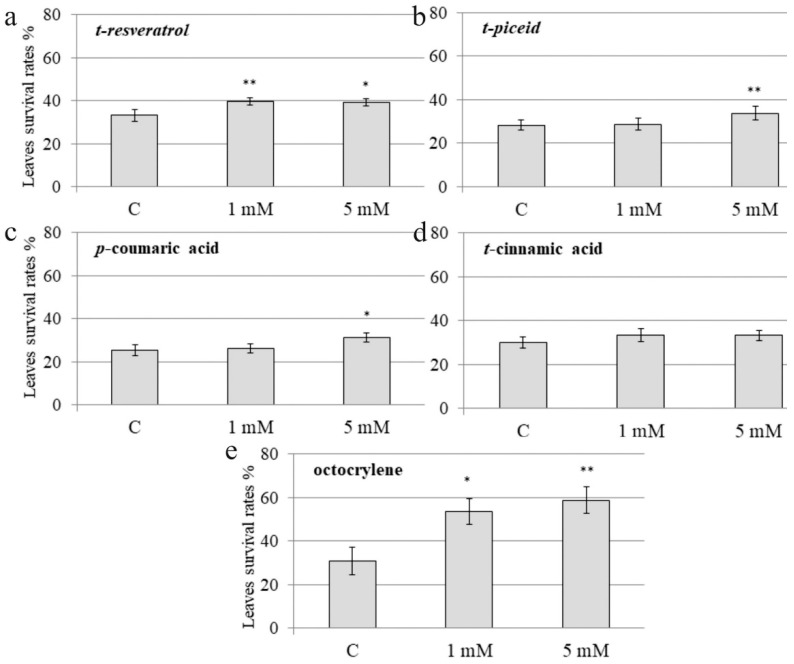
The effect of exogenous stilbenes, stilbene precursors and octocrylene on the leaf survival of *Arabidopsis thaliana* exposed to ultraviolet B (UV-B) irradiation. The leaf surface of four-week-old *A. thaliana* rosettes was treated with *t-*resveratrol (**a**), *t-*piceid (**b**), *p-*coumaric acid (**c**), *t-*cinnamic acid (**d**), octocrylene (**e**) and then exposed to UV-B 12 h after the chemical treatments. UV-B (312 nm) was applied for 10 min at a distance of 15 cm above the pots and as described [33]. C: water-treated *A. thaliana* exposed to the same UV-B irradiation conditions. The leaf survival rates were determined as the number of visibly green leaves seven days after the UV-B exposure. The data are presented as mean ± standard error. The data were obtained from five biological replicates (*n* = 40). *, ** Significantly different from the control water-treated *A. thaliana* at *p* < 0.05 and 0.01, respectively, according to the Student’s *t* test.

The sprayed *A. thaliana* plants were subjected to UV-B irradiation 12 h after the foliar application of *t-*resveratrol, *t*-piceid, *p*-coumaric acid, *t*-cinnamic acid, and octocrylene. The leaf survival rates were assessed seven days after the UV-B irradiation procedure. Leaf survival was studied after the chemical treatments and UV-B irradiation revealed that foliar application of *t*-resveratrol markedly increased the leaf survival rates of *A. thaliana* at both concentrations. Application of *t*-resveratrol increased the leaf survival by 6–7% versus own control, i.e., the control water-treated *A. thaliana* (Figure 1a). We also observed a significant plant protective effect after exogenous application of *t-*piceid and *p-*coumaric acid, which was detected only at 5 mM of *t-*piceid and *p-*coumaric acid; this was less pronounced than for *t-*resveratrol (a 6–7% increase for *t-*resveratrol versus 3–4% increase for *t-*piceid and *p-*coumaric acid; Figure 1). *T-*cinnamic acid did not considerably affect survival of the leaves and improved the rates by 1–2% (Figure 1). Among all tested stilbenes or their precursors, *t*-resveratrol exhibited the greatest protective effect and improved leaf survival at both concentrations. At the same time, we noted that the stilbene-induced leaf protection was lower than leaf protection after treatment with octocrylene, a compound widely used as a UV-B absorber (Figure 1).

### 2.3. Detection of t-Resveratrol on the A. thaliana Leaves after UV-B Treatment

**Figure 2:**
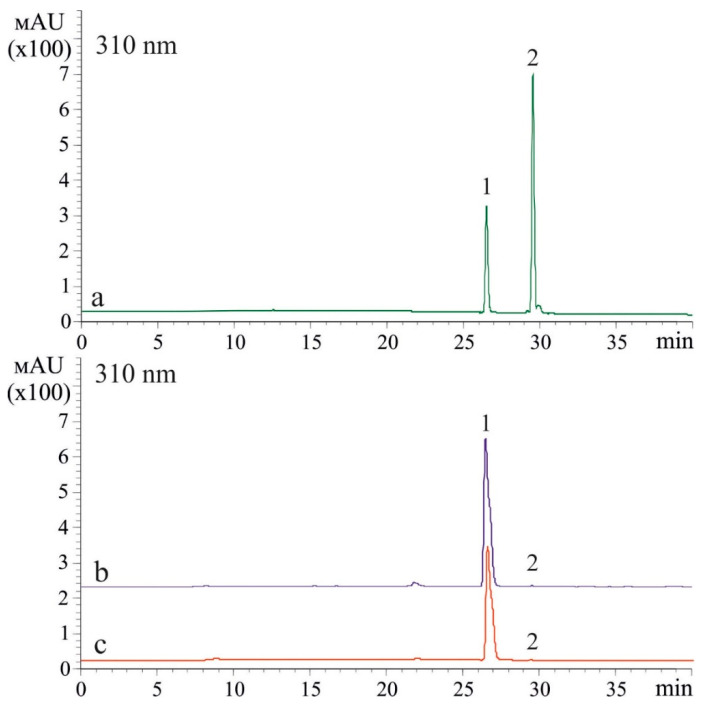
A representative HPLC profile (310 nm) of *t-*resveratrol and *cis-*resveratrol standards (**a**) and stilbenes extracted 2 h (**b**) and 12 h (**c**) after ultraviolet B (UV-B)- and *t*-resveratrol treatments of *Arabidopsis thaliana*. *Trans*-resveratrol (1) and *cis*-resveratrol (2).

The *t*-resveratrol may be metabolized to form other stilbenes [22], and thus there is a need to analyze whether the stilbene-induced UV-B protection effect might be caused by the presence of other stilbenes. Therefore, we analyzed the composition of stilbenes after application of *t*-resveratrol at 5 mM and UV-B irradiation (2 and 12 h after UV-B irradiation). We found that after the *t-*resveratrol and UV-B treatments, *t-*resveratrol was the major stilbene in the samples while *cis-*resveratrol appeared in trace amounts. We did not detect any other stilbenes (Figure 2). Nevertheless, we repeated this experiment three times and the proportion of *cis*-resveratrol has always been in traces.

### 2.4. The Effect of t-Resveratrol and UV-B Treatments on the Expression of Selected Stress-Inducible and DNA Repair Genes

**Figure 3:**
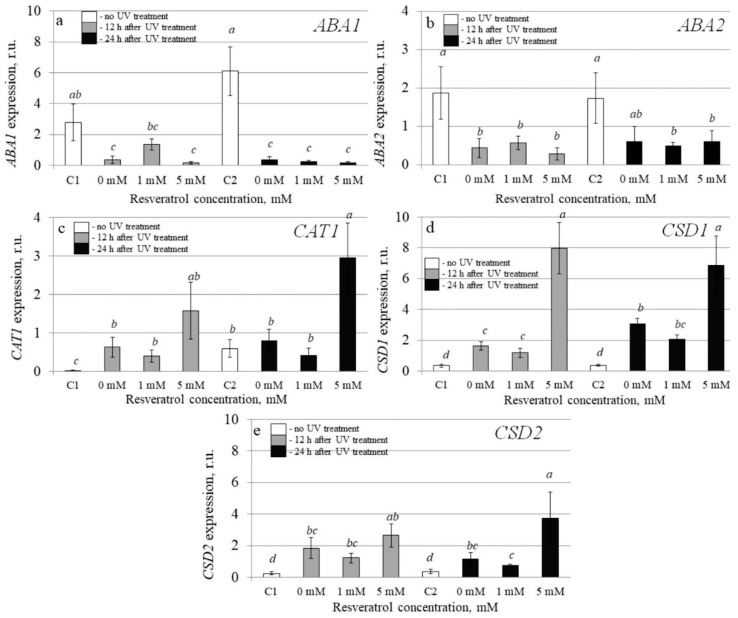
The effect of *t*-resveratrol and UV-B treatments on the expression of selected stress-responsive genes of *Arabidopsis thaliana*. The expression levels of ABA biosynthesis genes ((**a**), *ABA1*; (**b**), *ABA2*) and antioxidant genes ((**c**), *CAT1*; (**d**), *CSD1*; (**e**), *CSD2*) were analyzed. The four-week-old *A. thaliana* rosettes were sprayed with *t-*resveratrol and then exposed to UV-B 12 h after application of *t*-resveratrol. RNA was isolated before treatments, 12 h and 24 h after UV-B irradiation. C1 and C2: control *A. thaliana* not exposed to UV-B irradiation and not treated with *t-*resveratrol; 0: water-treated *A. thaliana* plants exposed to UV-B irradiation; 1 and 5: *t*-resveratrol-treated *A. thaliana* plants exposed to UV-B irradiation; r.u.–relative units. Means followed by the same letter were not different at *p* ≤ 0.05 using Student’s *t* test (three independent experiments).

**Figure 4:**
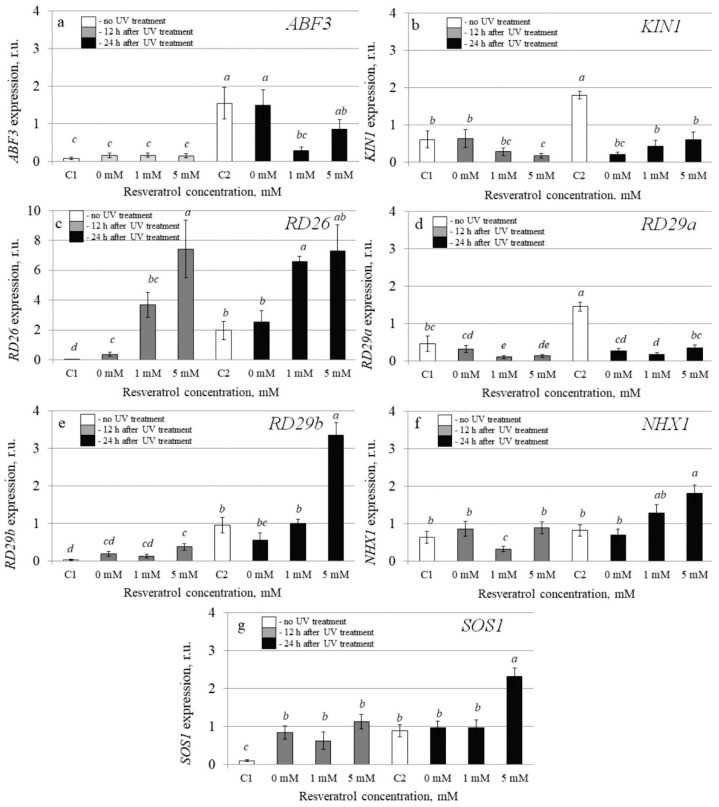
The effect of *t*-resveratrol and UV-B treatments on the expression of selected stress-responsive genes of *Arabidopsis thaliana*. The expression levels of stress-inducible regulatory genes ((**a**), *ABF3*; (**b**), *KIN1*, (**c**), *RD26*; (**d**), *RD29a*; (**e**), *RD29b*) and ion transporter genes ((**f**), *NHX1*; (**g**), *SOS1*) was analyzed. The four-week-old *A. thaliana* rosettes were sprayed with *t-*resveratrol and then exposed to UV-B 12 h after application of *t*-resveratrol. RNA was isolated before treatments, 12 h and 24 h after UV-B irradiation. C1 and C2: control *A. thaliana* not exposed to UV-B irradiation and not treated with *t-*resveratrol; 0: water-treated *A. thaliana* plants exposed to UV-B irradiation; 1 and 5: *t*-resveratrol-treated *A. thaliana* plants exposed to UV-B irradiation; r.u.–relative units. Means followed by the same letter were not different at *p* ≤ 0.05 using Student’s *t* test (three independent experiments).

**Figure 5:**
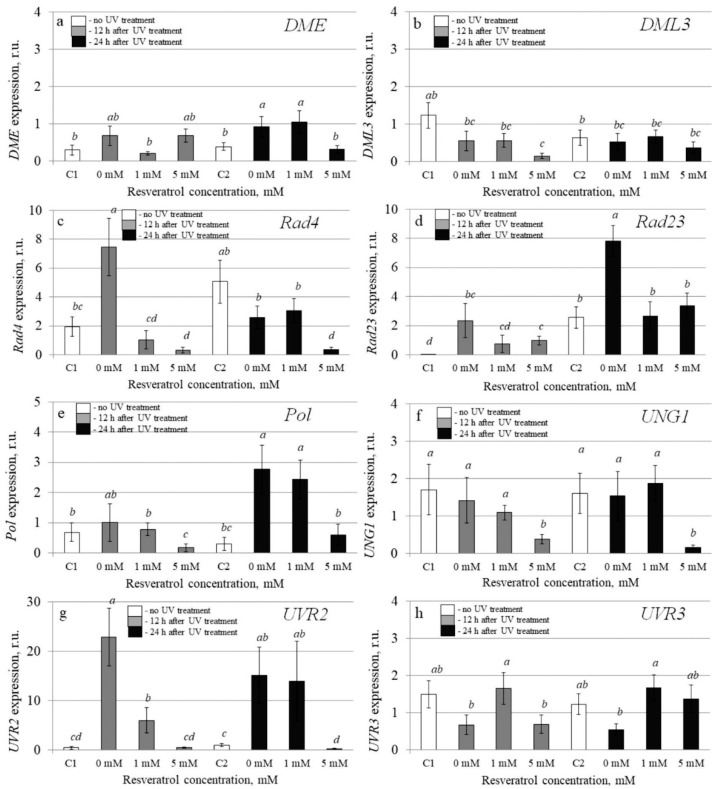
The effect of *t*-resveratrol and UV-B treatments on the expression of selected DNA repair genes of *Arabidopsis thaliana*. The expression levels of DNA demethylases ((**a**), *DME*; (**b**), *DML3*), DNA repair proteins ((**c**), *Rad4*; (**d**), *Rad23*), DNA polymerase ((**e**), *Pol*), DNA glycosylases ((**f**), *UNG1*), and photolyase ((**g**), *UVR2*; (**h**), *UVR3*) was analyzed. The four-week-old *A. thaliana* rosettes were sprayed with *t-*resveratrol and then exposed to UV-B 12 h after application of *t*-resveratrol. RNA was isolated before treatments, 12 h and 24 h after UV-B irradiation. C1 and C2: control *A. thaliana* not exposed to UV-B irradiation and not treated with *t-*resveratrol; 0: water-treated *A. thaliana* plants exposed to UV-B irradiation; 1 and 5: *t*-resveratrol-treated *A. thaliana* plants exposed to UV-B irradiation; r.u.–relative units. Means followed by *the same letter* were not different at *p* ≤ 0.05 using Student’s *t* test (three independent experiments).

To investigate the molecular mechanisms implicated in the resveratrol’s UV-B protective effect, we analyzed transcription levels of 20 stress-inducible *A. thaliana* genes by qRT-PCR in the control and *t-*resveratrol-treated *A. thaliana*. We evaluated the expression of five stress-responsive genes (*AtABF3*, *AtKin1*, *AtRD26*, *AtRD29A*, and *AtRD29B*), two ion transporter genes (*AtNHX1* and *AtSOS1*), two abscisic acid (ABA) biosynthesis genes (*AtABA1* and *AtABA2*), and three antioxidant genes (*AtCAT1*, *AtCSD1*, and *AtCSD2*). We also analyzed the expression of DNA repair genes including DNA glycosylase (*AtUNG1*), DNA polymerase (*AtPol*), DNA demethylase (*AtDML3* and *AtDME*), photolyase (*AtUVR2* and *AtUVR3*), and DNA repair proteins (*AtRad4* and *AtRad23a*) in the water- and *t-*resveratrol-treated *A. thaliana* exposed to UV-B irradiation and in the control plants not treated with UV-B (Figure 3, Figure 4 and Figure 5).

The data revealed that that the UV-B treatment greatly increased expression of the antioxidant genes including catalase 1 (*AtCAT1*), chloroplastic copper/zinc superoxide dismutase (*AtCSD1*), and cytosolic copper/zinc superoxide dismutase (*AtCSD2*) (Figure 3c–e). The *t-*resveratrol further enhanced expression of the *AtCAT1, AtCSD1*, and *AtCSD2* genes (Figure 3c–e). This study shows that *t-*resveratrol intensified this UV-based inducible effect on expression of antioxidant genes.

The analysis revealed that expression of the *AtRD26* gene dramatically increased after application of *t-*resveratrol (Figure 4c). We also observed a considerable increase in expression of ion transporter genes *AtNHX1* and *AtSOS* (vacuolar and plasma membrane Na^+^/H^+^ antiporters, respectively) induced by the foliar *t*-resveratrol application although to a lesser extent (Figure 4f,g). Furthermore, *t-*resveratrol strongly increased the expression of the gene known as responsive to dehydration 29B *(RD29b)*, which is a stress-inducible hydrophilic protein (Figure 4e). However, the expression of other analyzed genes (*AtABA1*, *AtABA2*, *AtABF3*, and *AtRD29a*) did not change after treatment with *t-*resveratrol or even decreased (*AtKIN1*) (Figure 3a,b and Figure 4a,b,d).

Further analysis revealed that UV-B treatment dramatically increased the expression of the *AtRad23* and *AtUVR2* 12 and 24 h post-treatment, but application of *t-*resveratrol removed this increase (Figure 5d,g). Moreover, the expression of almost all studied DNA repair genes decreased during treatment with *t-*resveratrol (Figure 5). UV-B irradiation considerably increased the expression of the *AtDME*, *AtRad4*, and *AtPol* genes at least at one time point. In this case, the addition of *t-*resveratrol reduced this elevation (Figure 5a,c,e). UV-B irradiation did not considerably affect the expression of the *AtDML3* and *AtUNG1* genes, but *t-*resveratrol treatment also had a down-regulating effect on their transcription levels (Figure 5b,f).

### 2.5. The Effect of Exogenous p-Coumaric Acid on the Content of Phenolic Secondary Metabolites in A. thaliana

**Figure 6:**
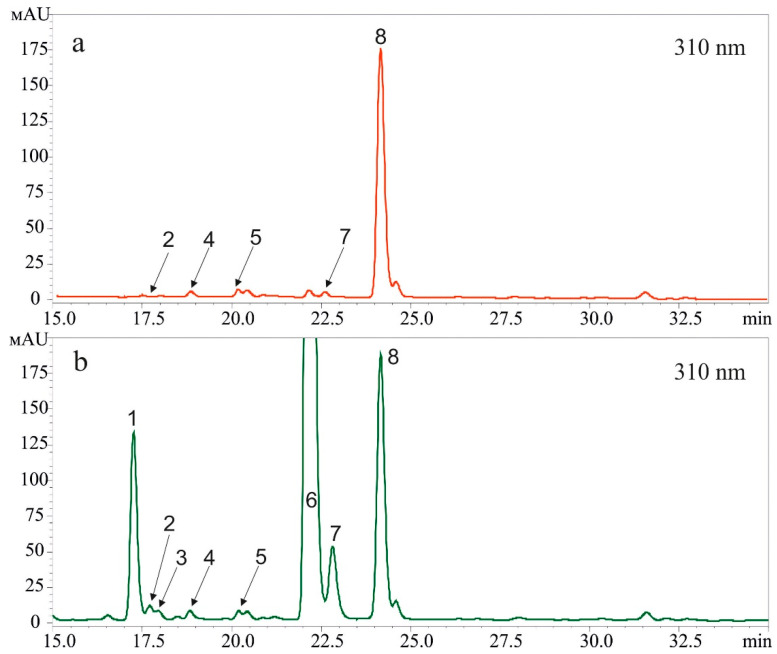
A representative HPLC-UV (310 nm) profile of the methanol extracts from *Arabidopsis thaliana* plants before (**a**) and 12 h after 5 mM *p*-coumaric (**b**). 1: coumaroyl hexoside (isomer 1); 2: glucohirsutin; 3: coumaroyl hexoside (isomer 2); 4: sinapoyl hexoside; 5: robinin; 6: *p-*coumaric acid; 7: kaempferol-3-O-glucosid-7-O-ramnoside; 8: sinapoyl malate.

It is possible that the exogenous stilbene precursor, *p-*coumaric acid, could increase the content of *A. thaliana*-derived phenolics and, thus, contribute to the UV-B protective effect of stilbene precursors. We analyzed the repertoire of *Arabidopsis*-derived phenolic secondary metabolites before treatment and 12 h after treatment with exogenous *p-*coumaric acid (Figure 6; Table 2). We analyzed the main peaks detected in the methanol-extracted samples and recorded values at 310 nm, i.e., phenolics, which possess UV-protective properties for *Arabidopsis* [34]. As a result, we determined five phenolic compounds before treatment with *p-*coumaric acid and seven compounds after treatment with *p-*coumaric acid (Figure 6; Table 2). Two new compounds that appeared after *p-*coumaric acid foliar application were *p-*coumaric acid and its glycosylated derivative, coumaroyl hexoside. These data indicate that plants can glycosylate a significant part of *p-*coumaric acid (about 20%) 12 h post-treatment. It is known that plant tissues metabolize the excess of any secondary metabolites by conjugation with carbohydrate residue [35].

Five defined substances were found before treatment with *p-*coumaric acid and are well-known *Arabidopsis* phenylpropanoid metabolites: glucohirsutin: 8-(methylsulfinyl)octyl-glucosinolate (2); sinapoyl hexoside: glycosylated derivative of sinapinic acid or sinapic acid, hydroxycinnamic acid, one of the phenolic acids, a member of the phenylpropanoid family (4); robinin, a flavone glycoside based on kaempferol (kaempferol-3-O-rhamnosyl-glucoside-7-O-rhamnoside (5); kaempferol-3-O-glucosid-7-O-ramnoside (7); and sinapoyl malate, is dicarboxylate anion of sinapic acid (S)-malate ester (8). Plant foliar treatment with *p-*coumaric acid markedly increased the content of all these compounds (Table 2, Figure 6), and the highest positive effect was detected for kaempferol-3-O-glucosid-7-O-rhamnoside. Kaempferol-3-O-glucosid-7-O-rhamnoside is a flavonol whose precursor is *p-*coumaric acid.

## 3. Discussion

In recent years, an increase in the solar UV-B radiation has been increasingly seen due to depletion of the ozone shield [1]. Sunlight is obligatory for photosynthesis, and the enhanced exposure to UV-B is especially detrimental to plants and interferes with plant survival. Plant phenolics accumulating in the epidermal layer of plant leaves are known to form a protective shield for the plant photosynthetic apparatus against ultraviolet radiation [36].

This study first shows that plant foliar treatments with stilbenes and stilbene precursors (primarily *t*-resveratrol) exhibited a plant protective effect against UV-B radiation improving leaf survival after excessive UV-B doses. The number of live green leaves was 6–7% higher after exogenous application of *t*-resveratrol than in control plants. At the same time, exogenous stilbenes did not affect the number of leaves and fresh weight of the *A. thaliana* rosettes. On the contrary, *p*-coumaric acid, a stilbene precursor, increased *A. thaliana* fresh weight. It is known that secondary metabolism competes with primary metabolism, which is responsible for the growth and development of plants. The competition is due to a requirement of similar energy sources and building materials. The external treatment with *p*-coumaric acid resulted in an abundance of some precursors, and this could reduce the load on the primary metabolism and increase plant growth. In other words, it is possible that *p*-coumaric acid was used by the plants as a precursor for the synthesis of some secondary metabolites or as a carbon source; therefore, it exhibited a growth-promoting effect. While the data suggested that foliar application of stilbenes and stilbene precursors led to a marked UV-protective effect, we noted that the stilbene-induced leaf protection was lower than leaf protection after treatment with octocrylene, a compound widely used as a UV-B absorber.

The *t*-resveratrol is known to convert into its *cis*-isomer (*cis*-resveratrol) under UV-B irradiation [37,38]; *t*-resveratrol may also metabolized to form other stilbenes such as pterostilbene, piceid, or viniferins [22]. Therefore, the observed stilbene-induced UV-B protection effect might be caused by the presence of the resveratrol isomer or other stilbenes. Thus, we analyzed the composition of stilbenes after application of *t*-resveratrol at 5 mM and UV-B irradiation (2 and 12 h after UV-B irradiation). We concluded that the detected UV-B protective effect on leaf survival resulted from application of *t*-resveratrol because this compound was the major stilbene in the samples after plant treatments with *t*-resveratrol and UV-B; *cis*-resveratrol appeared in trace amounts. We did not detect presence of any other stilbenes. Perhaps this was due to the fact that the UV-B irradiation was applied for only 10 min. In addition, we irradiated *t-*resveratrol-treated leaves, not pure *t*-resveratrol compound, while the published studies that described the conversion of *t-*resveratrol to *cis*-resveratrol [37,38] reported on a longer treatment and used a pure *t*-resveratrol compound.

*T*-resveratrol had the highest UV-B protective effect relative to other phenolic compounds (*t*-piceid, *p*-coumaric acid, *t*-cinnamic acid). Therefore, we decided to investigate the molecular mechanisms implicated in the resveratrol protective effect by analyzing transcription levels of a number of stress-inducible, antioxidant, ion transporter, abscisic acid biosynthesis-related, and DNA repair genes in *A. thaliana*. The data revealed that that the UV-B treatment greatly increased expression of the antioxidant genes including *AtCAT1*, *AtCSD1*, and *AtCSD2* while *t-*resveratrol further enhanced their expression. Catalase encoded by *CAT1* is a common enzyme catalyzing the decomposition of hydrogen peroxide to water and oxygen. Superoxide dismutase (*CSD1 CSD2)* is an enzyme that catalyzes the dismutation or partitioning of the superoxide (O^−2^) radical into ordinary molecular oxygen (O_2_) and hydrogen peroxide (H_2_O_2_) [39]. In general, it is well-known that the activity and expression of plant antioxidant genes strongly increases in response to UV-B irradiation [40].

The analysis revealed that expression of the *AtRD26, AtNHX1*, *AtSOS, RD29A*, and *RD29B* genes markedly increased after application of *t-*resveratrol. *AtRD26* is a NAC transcription factor whose expression was induced in response to drought and high salinity [41]. Overexpression of *NHX1* (a vacuolar Na^+^/H^+^ antiporter) in *Arabidopsis* plants promoted sustained plant growth and development in the soil watered with sodium chloride [42]; enhanced photoprotection was seen under high salinity and drought conditions [43]. Cold, drought, and salt induced both *RD29A* and *RD29B*. The *RD29A* promoter was more responsive to drought and cold stresses, whereas the promoter of *RD29B* was highly responsive to salt stress [44,45]. The expression of other analyzed genes (*AtABA1*, *AtABA2*, *AtABF3*, and *AtRD29a*) did not change after treatment with *t-*resveratrol, or even decreased (*AtKIN1*). This suggests that *t-*resveratrol caused a selective effect on the defense mechanisms in *A. thaliana*. Further analysis of DNA repair genes revealed that while UV-B irradiation enhanced expression of *AtRad23*, *AtUVR2, AtDME*, *AtRad4*, and *AtPol* genes, *t-*resveratrol treatment had a pronounced down-regulation effect on their transcript levels.

Coumaric acid is a known precursor for a wide range of phenolic secondary metabolites [46,47,48]. Therefore, it is possible that exogenous *p-*coumaric acid could increase the content of *A. thaliana* phenolics that are known to possess protective properties against UV-B light and, thus, contribute to the stilbene precursor UV-B protective effect. To test this hypothesis, we analyzed the repertoire of *Arabidopsis*-derived phenolic secondary metabolites before treatment and 12 h after treatment with exogenous *p-*coumaric acid. Plant foliar treatment with *p-*coumaric acid markedly increased the content of all compounds, and the highest positive effect was detected for kaempferol-3-O-glucosid-7-O-rhamnoside. Kaempferol and its derivatives are known to have a UV-B protective effect [37,49]. It is possible that this increase in the content of the endogenous protective phenolic metabolites of *A. thaliana* contributed to the improved resistance against UV-B- and *p-*coumaric acid-treated plants. We found that *t-*cinnamic acid increased *Arabidopsis* resistance to UV-B but to a lesser extent than other stilbenes and stilbene precursors; thus, it is possible that *t-*cinnamic acid was metabolized to other secondary metabolites in *A. thaliana*.

In summary, plant external treatments with stilbenes and stilbene precursors (primarily *t*-resveratrol) led to plant protective effects against UV-B radiation. The data obtained also indicated that transcriptional activation of a number of protective and antioxidant genes contributed to the UV-B protective effect of *t*-resveratrol. Therefore, *t-*resveratrol could be considered an effective UV-B protector. The results also show that the presence of stilbenes in plant tissues constitutes an effective mechanism for mitigating the UV-B damage and forming a protective shield against excessive UV-B radiation.

## 4. Materials and Methods

### 4.1. Plant Material and Growth Conditions

Five seven-day-old seedlings *Arabidopsis thaliana* ecotype Columbia L. (stored by our lab), pre-cultured on half-strength Murashige and Skoog (MS) medium with 0.8% agar, were planted in each individual pot (7 × 7 cm) filled with commercially available rich soil (100 g in each pot) in a controlled environmental chamber fixed at + 22 °C (KS-200, Smolenskoye SKTB SPU, Smolensk, Russia) kept on a 16/8 h day/night cycle at a light intensity of ~120 μmol m^−2^ s^−1^ [50].

### 4.2. Foliar Plant Chemical Treatments and UV-B Stress Tolerance Assays

Three groups of compounds were used for foliar plant treatments, including: (1) stilbenes (*t-*resveratrol and *trans*-piceid or *t-*piceid, *t-*resveratrol glycoside), (2) precursors of stilbenes and other phenolics (*p-*coumaric acid and *t-*cinnamic acid), and (3) octocrylene, which is a commercially used UV-B absorber. All used compounds were purchased in Sigma-Aldrich (Sigma, St. Louis, CA, USA). The compounds were firstly dissolved in dimethyl sulfoxide (DMSO) at a concentration of 1 M and then diluted in distilled water to 1 and 5 mM. For both the control and chemical treatments, we added 10 µL of DMSO per 2 mL of solution. Then, 2 mL of 1 and 5 mM aqueous solutions of *t-*resveratrol, *trans*-piceid (or *t*-piceid), *p*-coumaric acid, *trans*-cinnamic acid (or *t*-cinnamic acid), and octocrylene were sprayed with 2 mL atomizer polypropylene vials onto the adaxial and abaxial leaf surface of the four-week-old rosettes of *A. thaliana*. The plant chemical sprays were performed only once (12 h before application of UV-B stress). The *A. thaliana* plants were subjected to a shock of UV-B stress and assessed for the leaf survival rates seven days after the UV-B irradiation, as described by Ogneva et al. [29]. Briefly, four pots with four week-old *A. thaliana* (20 plants in total, five plants in each pot) were exposed to UV-B (312 nm) using a UV lamp VL-215.MC provided by Vilber Lourmat company (Vilber Lourmat Vilber Lourmat, Marne-la-Vallee, France). The plants were irradiated for 10 min at a distance of 15 cm above pots as according to Tyunin and Kiselev [33]. According to the manufacturer’s manual, we used 1800 µW cm^−2^ of UV-B irradiation intensity. The leaf survival rates were determined as the number of visibly green leaves seven days after the treatments.

### 4.3. High Performance Liquid Chromatography with Diode Array Detection (HPLC-DAD)

Total stilbene content on the leaves of the *A. thaliana* plants was measured by HPLC-DAD as described by Aleynova et al. and Kiselev et al. [48,51]. Briefly, 100 mg fresh leaves of *A. thaliana* were ground using a mortar and pestle in 1 mL of 1% (v/v) hydrochloric acid in methanol, and were extracted for 12 h at 10 °C in the dark. Samples were filtered through a Discovery® DSC-18 SPE Tube bed wt. 50 mg, volume 1 mL (Supelco, Bellefonte, PA, USA) and then used for HPLC-DAD analysis.

Identification of all compounds was performed using a 1260 Infinity analytical HPLC-DAD system (Agilent Technologies, Santa Clara, CA, USA), coupled to Bruker HCT ultra PTM Discovery System (Bruker Daltonik GmbH, Bremen, Germany), equipped with an electrospray ionisation (ESI) source. Data for all components of extracts were acquired in negative ions mode under the operating conditions as according to Kiselev et al. [51]. The MS spectra were recorded across an *m/z* range of 100–1500.

HPLC–DAD for quantification of all compounds was performed using a HPLC LC-20AD XR analytical system (Shimadzu, Kyoto, Japan). DAD data were recorded in the 200 nm–800 nm range, and chromatograms for quantification were acquired at 310 nm. The chromatographic separation was performed on Shim-pack GIST C18 column (150 mm, 2.1-nm i.d., 3-µm part size; Shimadzu, Japan). Extracts from *A. thaliana* were separated using 0.1% aqueous acetic acid and acetonitrile as mobile phases A and B, respectively, with the following elution profile: 0 to 35 min 0% of B; 35 to 40 min 40% of B; 40 to 50 min 50% of B; 50 to 65 min 100% of B. 3 μL of the sample extract was injected with a constant column temperature maintained at 40 °C and a flow rate maintained at 0.2 mL/min. All determined components of the extracts were identified as described by Kiselev et al. [37] on the base of UV spectra, recorded with a DAD detector, mass spectral data and chromatographic separation with reference to the values of their respective standards. The contents of each component were determined by using external standard method using the fives point regression calibration curves built with the reference standards.

The analytical standards: *trans*-resveratrol, *trans*-piceid, *trans*-piceatannol, *p*-coumaric acid and kaempferol were obtained from Sigma-Aldrich (Sigma, St. Louis, MO, USA). *d*-viniferin was obtained from Panreac AppliChem (GmbH, Darmstadt, Germany). *Cis* isomers of resveratrol and piceid were obtained under sunlight exposure of the respective standard solution containing the *trans*-isomer, as reported earlier by Kiselev et al. [51].

### 4.4. Total RNA Isolation and Real-Time Quantitative RT-PCR

Total RNA was isolated from the treated leaves of *A. thaliana* 12 and 24 h after UV-B exposure using the cetyltrimethylammonium bromide-based extraction as described by Kiselev et al. [52]. Complementary DNAs were synthesized using 1.5 µg of the total RNA by the RNA PCR Kit with SYBR Green I dye (Evrogen, Moscow, Russia) as according to Kiselev et al. [51]. cDNAs of *AtABA1*, *AtABA1*, *AtABF3*, *AtCAT1*, *AtCSD1*, *AtCSD2*, *AtDME*, *AtDML3*, *AtKIN1*, *AtNHX1*, *AtPOL*, *AtRad23*, *AtRad4*, *AtRD26*, *AtRD29a*, *AtRD29b*, *AtSOS1*, *AtUNG1*, *AtUVR2*, *AtUVR3*, *AtActin2*, and *AtGAPDH* genes were amplified using real-time PCR. The primers are listed in Table S1. The Gene Runner 5.0.78d software was used for primer design. The qRT-PCRs were performed using a Real-Time PCR Kit (Evrogen) in a thermocycler supplied with Multicolor Real-Time PCR Detection System (DNA Technology, Moscow, Russia). Expression was calculated using the 2^−ΔΔCT^ method [53]. *AtActin2* (GenBank NM_112764) and *AtGAPDH* (GenBank NM_111283) genes were used as endogenous controls to normalize variance in the quality and the amount of *A. thaliana* cDNA used in each qRT-PCR [51].

### 4.5. Statistical Analysis

Four pots with five four-week-old *A. thaliana* plants per each pot were treated in an independent experiment (20 *A. thaliana* plants in total). The data are presented as mean ± standard error (SE) and were tested by Student’s *t* test. The 0.05 level was selected as the point of minimal statistical significance in all analyses.

## Figures and Tables

**Table 1:** The effect of stilbenes (*t-*resveratrol, *t-*piceid), stilbene precursors (*p-*coumaric acid, *t-*cinnamic acid), and octocrylene (1 and 5 mM) on the growth and development of four-week-old wild-type *Arabidopsis thaliana*. The number of leaves and fresh rosette weight were calculated one week after the chemical treatments. The data were obtained from five biological replicates. * *p* < 0.05 versus values of control plants treated with water according to the Student’s *t* test.

Treatments	Number of Leaves in the Rosette	Fresh weight of the Rosette, FW
Control, H_2_0	13.96 ± 0.21	0.22 ± 0.02
DMSO	13.37 ± 0.42	0.22 ± 0.04
1 mM *t-*resveratrol	13.88 ± 0.61	0.22 ± 0.04
5 mM *t-*resveratrol	13.19 ± 0.45	0.19 ± 0.02
1 mM *t-*piceid	12.65 ± 0.67	0.22 ± 0.02
5 mM *t-*piceid	13.31 ± 0.51	0.27 ± 0.05
1 mM *p*-coumaric acid	14.33 ± 0.83	0.33 ± 0.05 *
5 mM *p*-coumaric acid	15.67 ± 1.02	0.29 ± 0.04
1 mM *t*-cinnamic acid	13.39 ± 0.45	0.23 ± 0.05
5 mM *t*-cinnamic acid	13.41 ± 0.46	0.24 ± 0.04
1 mM octocrylene	13.27 ± 0.43	0.21 ± 0.04
5 mM octocrylene	13.16 ± 0.39	0.21 ± 0.05

**Table 2:** The effect of 5 mM *p*-coumaric acid on the phenolic content (methanol extraction, HPLC-DAD recorded at 310 nm) in the four-week-old wild-type *Arabidopsis thaliana*. The data were obtained from three biological replicates. * *p* < 0.05 and ** *p* < 0.01 versus values in plants before *p*-coumaric treatment, according to the Student’s *t* test.

Peak Numbers ^a^	Compounds	Control, before Treatment, mg/g FW	5 mM *p*-Coumaric Acid, mg/g FW
1, 3	Coumaroyl hexoside (isomer 1 and 2)	0.0 ± 0.0	2.64 ± 0.64 **
2	Glucohirsutin	0.05 ± 0.03	0.57 ± 0.20 *
4	Sinapoyl hexoside	0.06 ± 0.03	0.17 ± 0.06 *
5	Robinin, kaempferol 3-O-rhamnosyl-glucoside 7-O-rhamnoside	0.32 ± 0.07	0.57 ± 0.13 *
*6*	*p-*coumaric acid	0.0 ± 0.0	10.32 ± 1.45 **
7	Kaempferol-3-O-glucosid-7-O-ramnoside	0.21 ± 0.07	5.26 ± 1.12 **
8	Sinapoyl malate	2.29 ± 0.47	3.96 ± 0.79 *

^a^ The peak numbers are shown as in the Figure 5.

## Supplementary Materials

The following are available online at https://www.mdpi.com/article/10.3390/plants10071282/s1, Table S1: Primers used for amplification of *Arabidopsis thaliana* cDNAs in real-time PCRs.
