# Supplementary material for: Exogenous Stilbenes Improved Tolerance of Arabidopsis thaliana to a Shock of Ultraviolet B Radiation
Source: Plants (Basel). 2021 Jun 24;10(7):1282. doi: 10.3390/plants10071282 (PMC8308955; doi:10.3390/plants10071282)
Supplement: Supplementary file 1 [file plants-10-01282-s001.zip › plants-1240744-supplementary.pdf]

Ogneva et al. 2021, Plants.

Supplementary Table S1.
Primers used for amplification of *Arabidopsis thaliana* cDNAs in real-time PCRs.

cDNA	Primers, 5’-3’
AtActin2 (NM_112764)	GATTCAGATGCCCAGAAGTC, TACCGTACAGATCCTTCCTG
AtGAPDH (NM_111283)	TTGGTGACAACAGGTCAAGCA, AAACTTGTCGCTCAATGCAAT
AtABA1 (NM_180954)	GCT ATG AAG GTG ATC TGC TTG TGG, TTC ATA CCA TTT GGA GCA TCA GC
AtABA2 (AY082345)	ATT GAT CAC TGG AGG AGC CAC AG, ATT ACG AAT ATC AGG GCA CGG TG
AtABF3 (NM_001036708)	CAA CAT CAG CAA TGG TAA TAG TGG A, CGT CCG AGG CAA GGT AAG TG
AtCAT1 (NM_101914)	AGC GCT TTC GGA GCC TCG TG, GGC CTC ACG TTA AGA CGA GTT GC
AtCSD1 (NM_100757)	GTT GGT AGG GCT GTT GTT GTC, TGG ACC TCC TTA TTA CAT CAA
AtCSD2 (NM_128379)	TTA GTC TGA CCA CTG GAA ACG C, GGA TGC TAA ATA AAC CAA AAT GTA
AtDME (NM_120538)	CGATGTTCCTAGAGATTGGAT, CAGGAAAATGCAACCTTGCC
AtDML3 (NM_119567)	GCTTAAACCCCATTGTCTTTC, TCTCATCAGGTGGAGTGTG
AtKIN1 AY114587	CCA ACA AGA ATG CCT TCC AAG C, GCT GCC GCA TCC GAT ACA CT
AtNHX1 (NM_122597)	CCG TGC ATT ACT ACT GGA GAC AAT, GTA CAA AGC CAC GAC CTC CAA
AtPol (DQ446242)	GACTTGACGACACTGGACTA, GTTTCTCCGTTGAGAGTTTCAG
AtRad23 (NM_101486)	CATAGAAGCCTTCCTTTC, GTCTTCAAAATCTGCTGAG
AtRad4 (BT010359)	GTAAGAAAGTCTCGTAATGAAGA, AGGTTTCCTCATCAAAGGT
AtRD26 (NM_118875)	GAT GTG AAG TTA CTG ATG GGT GAA, GCG AGC CAA GTC ACA AGG AG
AtRD29A (NM_124610)	ATC ACT TGG CTC CAC TGT TGT TC, ACA AAA CAC ACA TAA ACA TCC AAA G
AtRD29B (NM_124609)	GGA ATC CGA AAA CCC CAT AGT C, GGA GTG AAG GAG ACG CAA CAA G
AtSOS1 (NM_126259)	TCG TTT CAG CCA AAT CAG AAA GT, TTT GCC TTG TGC TGC TTT CC
AtUNG (BT029175)	ACGCTCAAGAGAAATCCAAGTT, GCTGGTTTGCGCGAGAGAAA
AtUVR2 (NM_101109)	GCAAACGGAAATTCAATGTGGA, CTTGGGATCAACAGAGTCTCTGG
AtUVR3 (NM_001035626)	CCATCTAGCGCGTCACTGT, GACGAACATGATAACCACATC
